# Supplementary material for: Room Temperature Control of Axial and Basal Antiferromagnetic Anisotropies Using Strain
Source: ACS Nano. 2025 Dec 10;19(50):42118–27. doi: 10.1021/acsnano.5c12282 (PMC12752703; doi:10.1021/acsnano.5c12282)
Supplement: Supplementary file 1 [file nn5c12282_si_001.pdf]

Supplementary Information

Room temperature control of axial and basal antiferromagnetic anisotropies using strain

Jack Harrison,[1,2,†] *Junxiong Hu,*[3,4] *Charles Godfrey,*[1] *Jheng-Cyuan Lin,*[1,5] *Tim A Butcher,*[2,6] *Jörg Raabe,*[2] *Simone Finizio,*[2] *Hariom Jani,*[1,†,*] *Paolo G Radaelli* [1]

[1] Clarendon Laboratory, University of Oxford, Oxford, OX1 3PU, UK
[2] Paul Scherrer Institute, 5232 Villigen PSI, Switzerland
[3] School of Physics, University of Electronic Science and Technology of China, 611731, Chengdu, China
[4] Department of Physics, National University of Singapore, 119077, Singapore
[5] Diamond Light Source, Didcot OX11 0DE, UK
[6] Max Born Institute for Nonlinear Optics and Short Pulse Spectroscopy, 12489, Berlin, Germany

[†] These authors contributed equally.

*Correspondence: hariom.jani@physics.ox.ac.uk;

S1 Experimental setup

Figure S1 contains a schematic of the gas cell used to flex the membranes. The inside of the cell was pressurised by a He gas input with flow rates 0-100 cc/min and a PID-controlled needle valve connected to a vacuum pump set and maintained the pressure in the range 0-800 mbar. The STXM chamber is held under vacuum. Valve V1 allows the cell to be connected to the STXM chamber, ensuring a zero-pressure differential state at the start of the experiment. Subsequently, the He gas is inserted into the cell through valve V2 and the pressure is controlled by varying the flow speed in and out of the chamber (with V1 disconnected). The pressure difference between the cell and the chamber results in controlled membrane flexure and, thereby, the application of systematic symmetric and asymmetric strain in square and rectangular membrane holders.

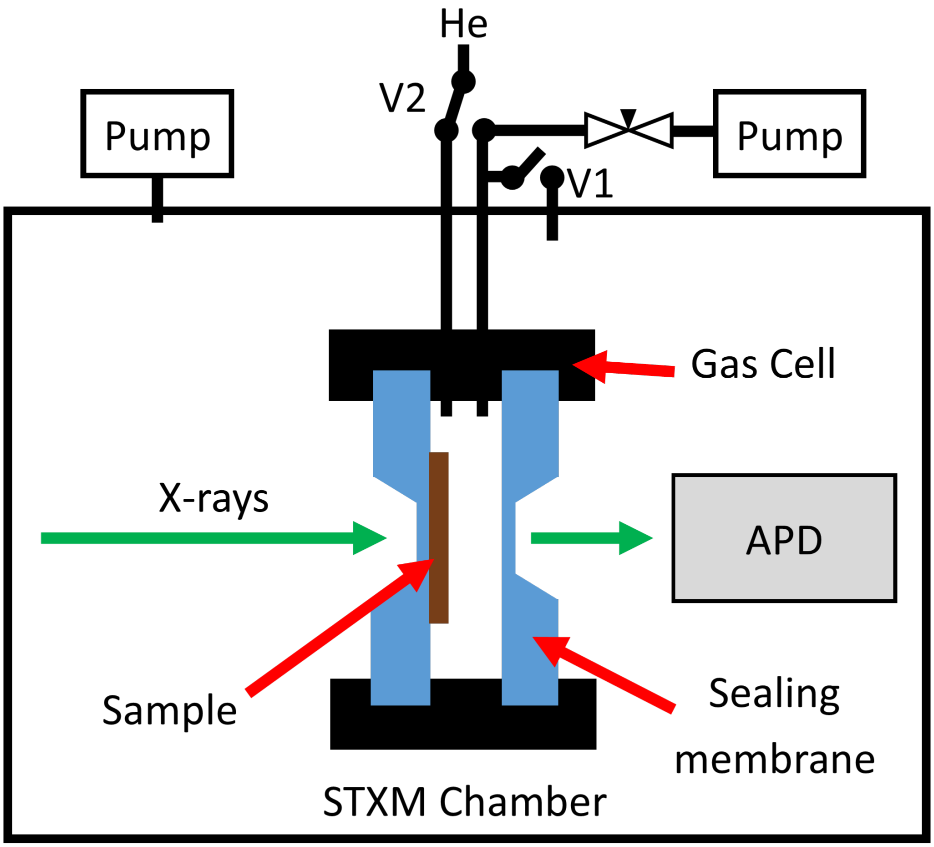

Figure S1: Schematic of the gas cell setup employed in our experiments.

S2 Morin transition hysteresis and reversibility under strain

To demonstrate that the thermal hysteresis inherent in the first-order Morin transition[1–3] is reproduced under strain, we took images thermally below the transition in an unstrained state, strained the membrane until we crossed through the transition, and then relaxed it back to the unstrained state. As shown in figure S2, the final state is an intermediate between the IP and OOP phases, demonstrating that there is the same hysteresis in the strain-induced Morin transition, as would be expected by the Landau model introduced in equations 1 and 2 (main text). When a similar pressure cycle is performed several times at a lower temperature, sufficiently below the

Morin transition, then the full OOP phase with IP domain walls is recovered, see figure S3. This demonstrates that this effect is truly hysteresis across a first order phase transition rather than any irreversible changes caused *e.g.* by plastic deformation of the membrane.

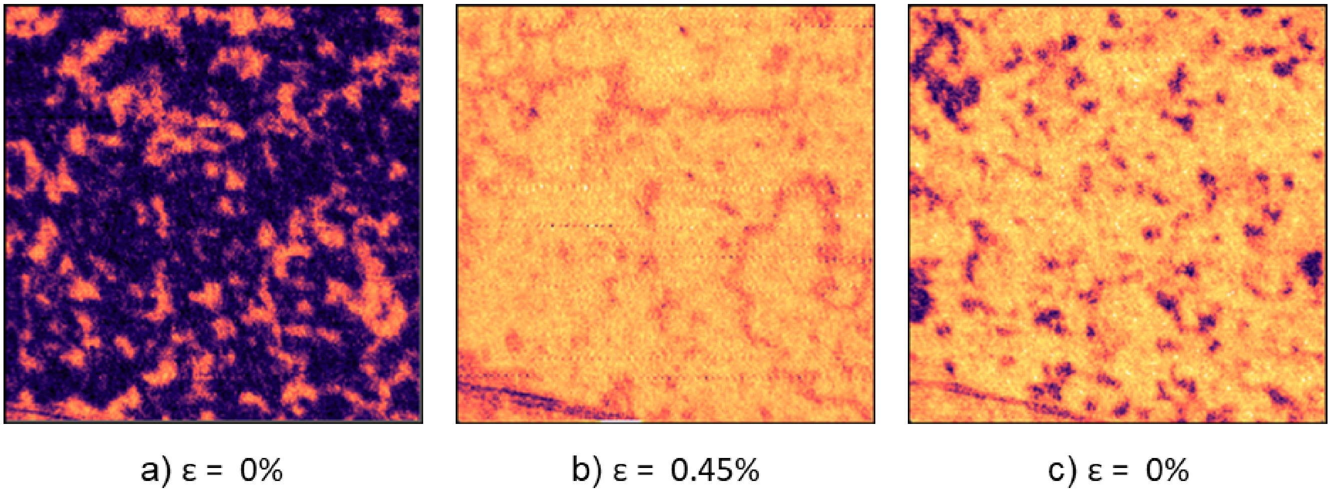

Figure S2: XMLD-STXM image taken at 306 K on a square membrane with an inherent (zero-strain) transition at 309 K. These images were taken in sequence and with an applied gas cell pressure of (a) 0 mbar, (b) 300 mbar, and (c) 0 mbar.

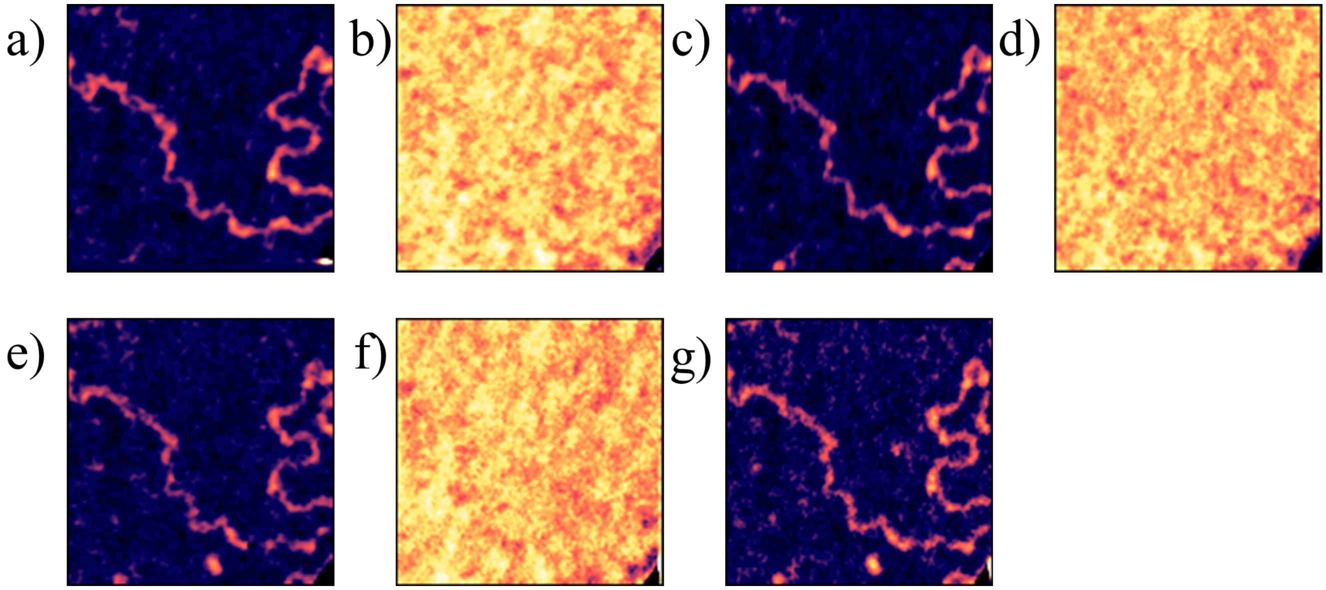

Figure S3: XMLD-STXM images taken at 298 K on a rectangular membrane with an inherent (zero-strain) transition at 310 K. These images were taken in sequence and with an applied gas cell pressure of (a,c,e,g) 0 mbar, (b,d,f) 700 mbar during the first (b,c), second (d,e), and fifth (f,g) pressurising/depressurising cycles.

S3 Angular distribution of domains in symmetrically strained membrane

As discussed in the main text, asymmetrically strained rectangular membranes result in a uniaxial redistribution of AFM order under strain. Contrarily, symmetrically strained square membranes

do not produce such in-plane uniaxial anisotropy. To confirm this, we generated a similar vector map and corresponding pole plot taken on a square membrane under biaxial strain, measured above the Morin transition. The pole plot is similar to that shown in the unstrained state on a rectangular membrane in figure 4a (main text), featuring approximately similar proportions of all in-plane orientations. This is clearly very different from the strongly uniaxial distribution in figures 4b,c (main text), and hence verifies that the strain-induced effects are indeed distinct in the cases of square and rectangular membranes.

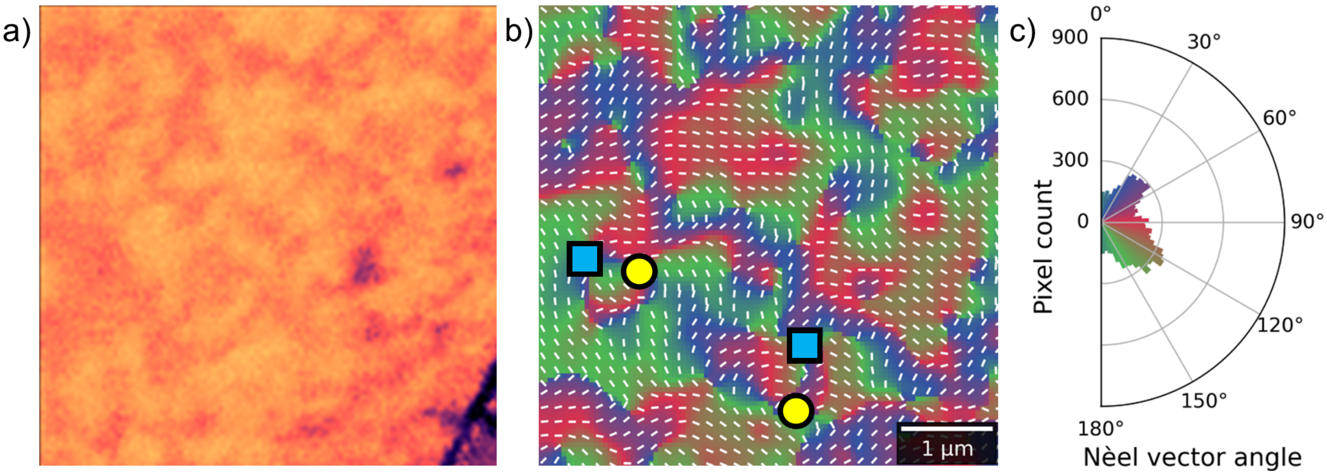

Figure S4: a) XMLD-STXM image taken at 280 K on a square membrane with an inherent (zero-strain) transition at 309 K with $\varepsilon = 0.45\%$, a repeat of figure 2d (main text). b) The vector map of the strained state shown in (a). c) corresponding pole plot, showing a domain distribution similar to the unstrained state in the rectangular membrane in figure 4a (main text).

S4 Strain-driven Morin transition in rectangular membranes

Figure S5 shows a set of images collected on a pressurised rectangular membrane at room temperature (299 K), under increasing strain. As this temperature is below T_{M}, the sample starts in the OOP phase (purple) separated by IP anti-phase domain walls (yellow/orange).[3–5] Straining the sample initially reproduces the same phenomenology as in the case with symmetric strain (figure 2, main text). Hence, both symmetric and asymmetric strain can suppress the axial anisotropy and thereby lower the effective T_{M}. This is consistent with the Landau model presented in equation 1.

S5 Magnetoelastic energy for α-Fe_2O_3

The magnetoelastic tensor of hematite is well-known and has the form[6,7]

$$\begin{aligned} F_{\mathrm{ME}} = {} & (\delta_1\gamma_x^2 + \delta_2\gamma_y^2 + \delta_5\gamma_y\gamma_z)\varepsilon_{xx} + (\delta_2\gamma_x^2 + \delta_1\gamma_y^2 - \delta_5\gamma_y\gamma_z)\varepsilon_{yy} + \delta_3\gamma_z^2\varepsilon_{zz} \\ & + (2(\delta_1 - \delta_2)\gamma_x\gamma_y + 2\delta_5\gamma_x\gamma_z + \delta_5\gamma_y\gamma_z)\varepsilon_{xy} \\ & + (2\delta_6\gamma_x\gamma_y + \delta_4\gamma_x\gamma_z)\varepsilon_{xz} + (\delta_6(\gamma_x^2 - \gamma_y^2) + \delta_4\gamma_y\gamma_z)\varepsilon_{yz} \end{aligned} \tag{S1}$$

where δ_i are the magnetoelastic constants of the material, $\gamma_{x,y,z} = (\sin\theta\cos\phi, \sin\theta\sin\phi, \cos\theta)$ are the directional cosines of the Néel vector, and ε_{ij} are the strain tensor coefficients. For this expression, the z-axis is defined as the threefold axis of the crystal structure and the x-axis is

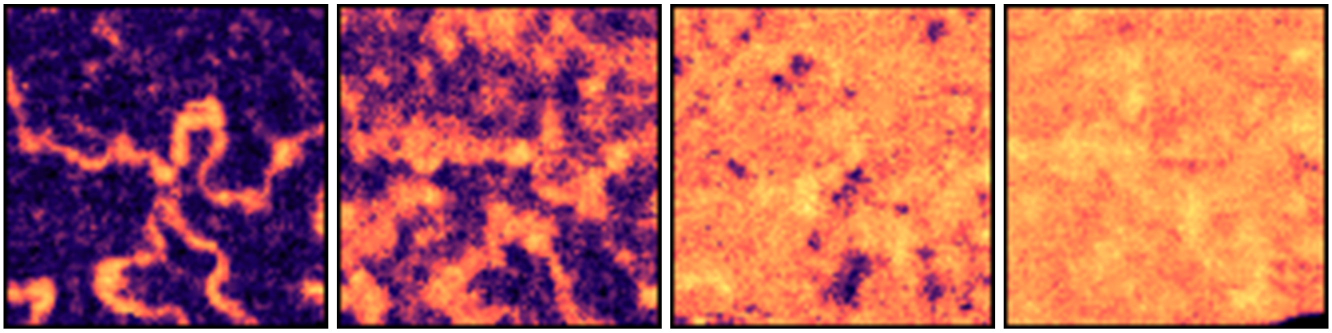

a) ε_{11} = 0%   b) ε_{11} = 0.84%   c) ε_{11} = 1.54%   d) ε_{11} = 3.11%

Figure S5: STXM XMLD image on a rectangular membrane at room temperature ($T < T_{\mathrm{M}}$) as a function of applied strain. The applied pressures were (a) 0 mbar, (b) 400 mbar, (c) 600 mbar and (d) 800 mbar. Purple and orange/yellow colours correspond to OOP and IP Néel vector respectively.

one of the twofold axes. For our membranes, this means the z-axis is out-of-plane and the x-axis corresponds to the b direction, which is the vertical axis relative to the shown images (*e.g.* figures 2, 4). Therefore $\varepsilon_{xx} = \varepsilon_{22}$ and $\varepsilon_{yy} = \varepsilon_{11}$. As shown in figures 1 and 3, main text, any shear components of the strain are very small compared to the diagonal components near the membrane centre. Ignoring these shear terms and substituting in the relevant angles $\gamma_{x,y,z}$ gives

$$\begin{aligned} F_{\mathrm{ME}} = & \frac{1}{2}\sin^2\theta\,[(\delta_1+\delta_2)(\varepsilon_{11}+\varepsilon_{22}) + (\delta_1-\delta_2)(\varepsilon_{22}-\varepsilon_{11})\cos 2\theta] \\ & + \delta_3\varepsilon_{33}\cos^2\theta + \delta_5\sin\theta\cos\theta\sin\phi(\varepsilon_{22}-\varepsilon_{11}). \end{aligned} \tag{S2}$$

We can relate the OOP component of strain ε_{33} to the in-plane components by utilising the stress-strain tensor for in-plane stress in -3m symmetry and ignoring shear components.[8] Assuming that the vertical component of stress $\sigma_{33} = 0$ under the planar membrane assumption gives the relation, $\varepsilon_{33} = \frac{-c_{13}}{c_{33}}(\varepsilon_{11}+\varepsilon_{22})$, where $\frac{-c_{13}}{c_{33}} \approx -0.4$.[6] Hence, we can substitute this into equation S2 to give

$$\begin{aligned} F_{\mathrm{ME}} = & \frac{1}{2}\sin^2\theta\,[(\delta_1+\delta_2+0.8\delta_3)(\varepsilon_{11}+\varepsilon_{22}) + (\delta_1-\delta_2)(\varepsilon_{22}-\varepsilon_{11})\cos 2\theta] \\ & - 0.4\delta_3(\varepsilon_{11}+\varepsilon_{22}) + \delta_5\sin\theta\cos\theta\sin\phi(\varepsilon_{22}-\varepsilon_{11}). \end{aligned} \tag{S3}$$

The second term in this equation is a purely elastic energy term and can be ignored when studying the magnetic structures. Furthermore, the final term is identically zero in both the easy-plane and easy-axis phases, so can be ignored. This equation is then equivalent to equation 2 (main text) with $K_{\mathrm{S1}} = \delta_1+\delta_2+0.8\delta_3$ and $K_{\mathrm{S2}} = \delta_1-\delta_2$.

S6 Micromagnetic simulations

Here, we expand on the approach to micromagnetic simulations, in particular focusing on the implementation of the basal plane anisotropy and strain-induced uniaxial anisotropy. The basic approach utilises our 'multi-cell' stack model for an A-type AFM.[9]

The basal plane anisotropy is of the form $K_B\cos^2(3\phi)$ (see main text, equation 2). For implementation in Mumax3,[10–12] this needs to be re-expressed as a function of products of the unit (sublattice) magnetisation $\vec{m}$ with some defined anisotropy axes. To do this, we use the expansion

$$\cos^2(3\phi) = 16\cos^2(\phi)\cos^2(\phi+\pi/3)\cos^2(\phi-\pi/3). \tag{S4}$$

If one then defines the three basal anisotropy easy-axes as $\vec{u}_1 = (1,0,0)$, $\vec{u}_2 = (-1/2, \sqrt{3}/2, 0)$, and $\vec{u}_3 = (-1/2, -\sqrt{3}/2, 0)$, then each of the three terms in the product above can be expressed in the form $\vec{u}_i \cdot \vec{m}$. The anisotropy must be implemented in Mumax3 as an effective field term,[10] which for the three components of our basal anisotropy gives

$$\vec{B}_i = \frac{32K_b}{M_s}\left((\vec{u}_i \cdot \vec{m})(\vec{u}_j \cdot \vec{m})^2(\vec{u}_k \cdot \vec{m})^2\right)\vec{u}_i, \tag{S5}$$

where $i,j,k = \{1,2,3\}$ runs over the three anisotropy axes. The total effective field is then the vector sum of these three contributions, i.e. $\vec{B}_{eff} = \vec{B}_1 + \vec{B}_2 + \vec{B}_3$.

To introduce a strain-driven uniaxial anisotropy into this system as in equation 3 (main text), we introduce an extra term of the form

$$\vec{B}_{\text{strain}} = K_\varepsilon(\vec{u}_s \cdot \vec{m})\vec{u}_s, \tag{S6}$$

where $K_\varepsilon = \frac{1}{2}K_{S2}(\varepsilon_{11} - \varepsilon_{22})$ determines the strength of the anisotropy and $\vec{u}_s$ defines the anisotropy axis. For the maximum value of induced anisotropy investigated here of $k_\varepsilon = 3\,\text{kJm}^{-3}$, roughly corresponding to the maximum strain state where $\varepsilon_{11} - \varepsilon_{22} = 1.2\%$, the associated magnetoelastic constant is $K_{S2} = 500\,\text{kJm}^{-3}$. This value is of the same order of magnitude as the magnetoelastic constants reported for hematite in the literature – approximately $800\,\text{kJm}^{-3}$ and $1700\,\text{kJm}^{-3}$.[7,13] Such deviations are entirely reasonable given that the strain coupling in freestanding membranes may differ from that of bulk crystals. Some key results for a meron initialization are shown in Figure 5 (main text) and figure S6.

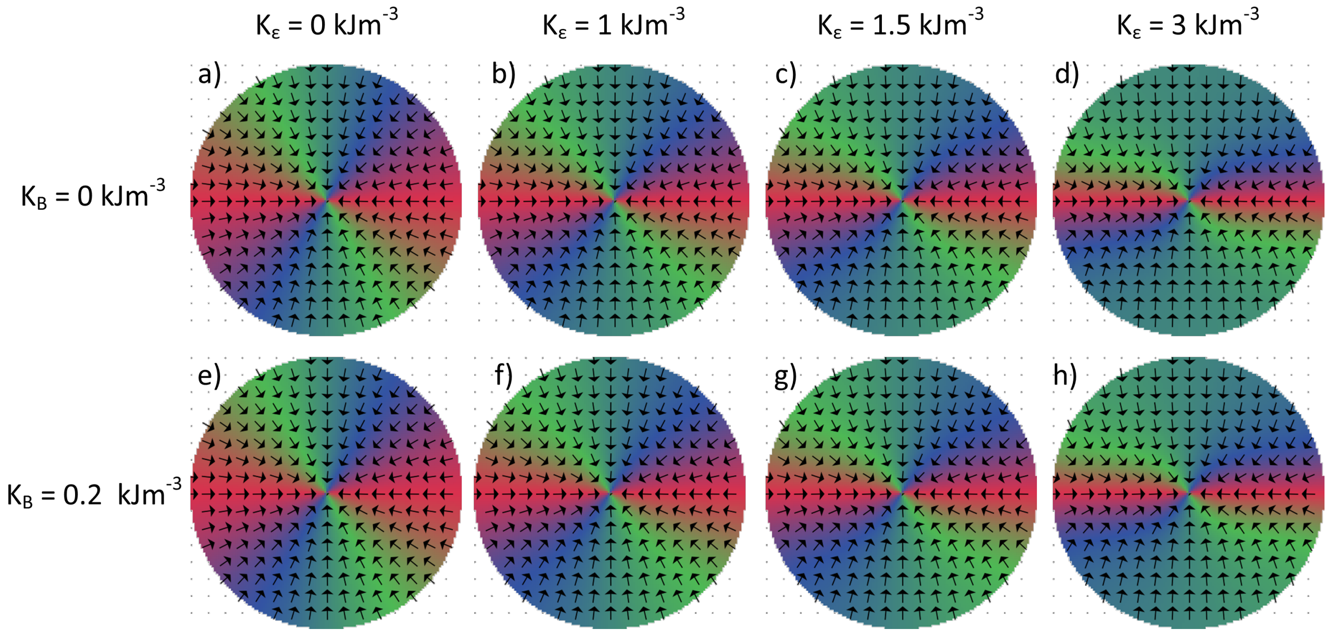

Figure S6: Simulated meron configurations as different values of corresponding to the pole plots in figure 5, main text.

S7 Vector map images

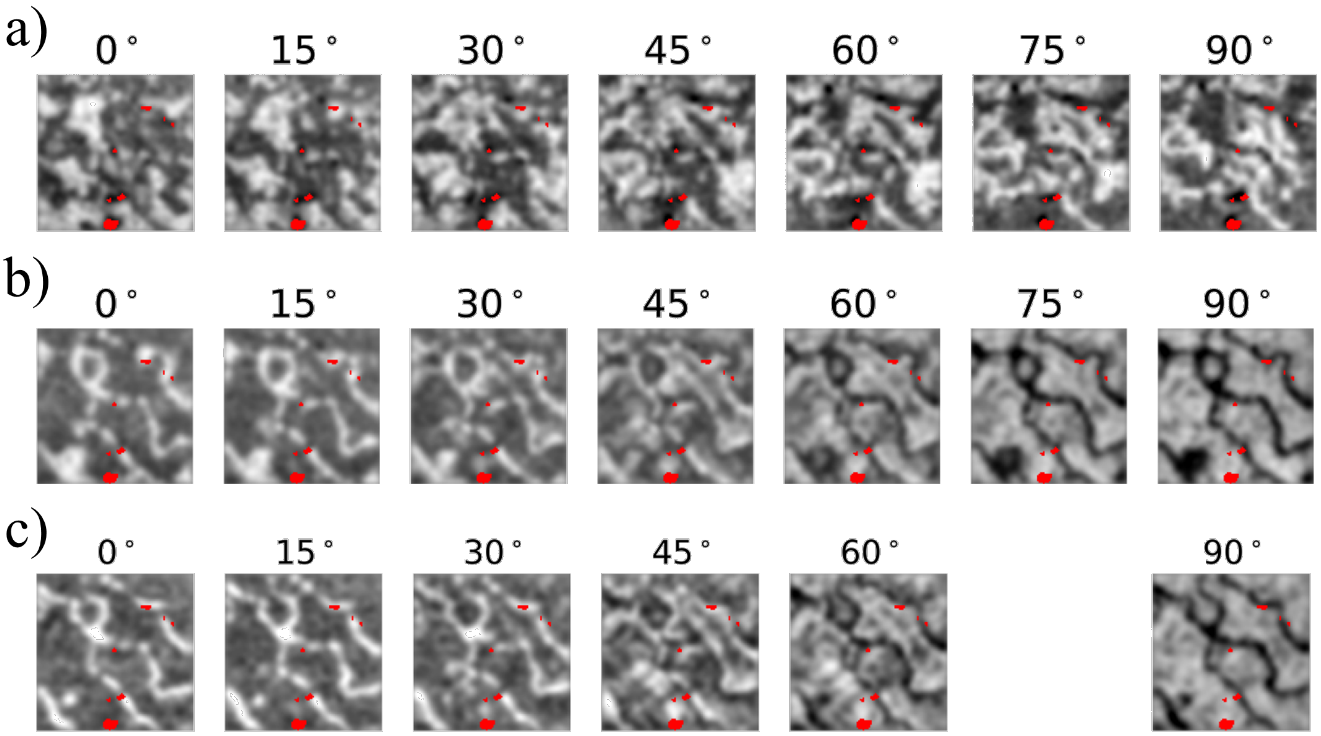

Figure S7: STXM XMLD greyscale images as a function of angle for the asymmetrically strained membranes at different applied pressures used to construct the vector maps in figure 4 (main text). a) 0 mbar, b) 400 mbar and c) 800 mbar. The red regions represent defects, which have been masked out. In (c), the 75° image was poor quality and so was excluded from the vector mapping and is not shown here.

References

[1] Morin, F. J. Magnetic Susceptibility of α-Fe_2O_3 and α-Fe_2O_3 with Added Titanium. *Physical Review* **1950**, *78*, 819–820.

[2] Coey, J. M. D. *Magnetism and Magnetic Materials*; Cambridge Universiy Press, 2009; pp 418–420.

[3] Jani, H.; Lin, J.-C.; Chen, J.; Harrison, J.; Maccherozzi, F.; Schad, J.; Prakash, S.; Eom, C.-B.; Ariando, A.; Venkatensan, T.; Radaelli, P. G. Antiferromagnetic Half-Skyrmions and Bimerons at Room Temperature. *Nature* **2021**, *590*, 74–79.

[4] Jani, H.; Harrison, J.; Hooda, S.; Prakash, S.; Nandi, P.; Hu, J.; Zeng, Z.; Lin, J.-C.; Godfrey, C.; Omar, G.; Butcher, T.; Raabe, J.; Finizio, S.; Thean, A. V.-Y.; Ariando, A.; Radaelli, P. Spatially Reconfigurable Antiferromagnetic States in Topologically Rich Free-Standing Nanomembranes. *Nature Materials* **2024**, *23*, 619–626.

[5] Harrison, J.; Jani, H.; Hu, J.; Lal, M.; Lin, J.; Popescu, H.; Brown, J.; Jaouen, N.; A., A.; Radaelli, P. Holographic Imaging of Antiferromagnetic Domains with In-Situ Magnetic Field. *Opt. Express* **2024**, *32*, 5885–5897.

[6] Morrish, A. H. *Canted Antiferromagnetism: Hematite*; World Scientific, 1994.

[7] Levitin, R. Z.; Pakhomov, A. S.; Shchurov, V. A. The Magnetoelastic Coupling Constants of Hematite. *Phys. Lett.* **1968**, *27*, 603.

[8] Nye, J. *Physical Properties of Crystals : Their Representation by Tensors and Matrices*; Oxford science publications; Clarendon Press, 2009.

[9] Harrison, J.; Jani, H.; Radaelli, P. G. Route Towards Stable Homochiral Topological Textures in A-Type Antiferromagnets. *Physical Review B* **2022**, *105*, 224424.

[10] Vansteenkiste, A.; Leliaert, J.; Dvornik, M.; Helsen, M.; Garcia-Sanchez, F.; Van Waeyenberge, B. The Design and Verification of MuMax3. *AIP Advances* **2014**, *4*, 107133.

[11] Exl, L.; Bance, S.; Reichel, F.; Schrefl, T.; Peter Stimming, H.; Mauser, N. J. LaBonte's Method Revisited: an Effective Steepest Descent Method for Micromagnetic Energy Minimization. *Journal of Applied Physics* **2014**, *115*, 17D118.

[12] Mulkers, J.; Van Waeyenberge, B.; Milošević, M. V. Effects of Spatially-Engineered Dzyaloshinskii-Moriya Interaction in Ferromagnetic Films. *Physical Review B* **2017**, *95*, 144401.

[13] Levitin, R. Z.; Pakhomov, A. S.; Shchurov, V. A. Magnetoelastic Properties of Hematite. *Sov. Phys. Jetp* **1969**, *29*, 1242–1251.
